# Sylvatic cycles of arboviruses in non-human primates

**DOI:** 10.1186/s13071-019-3732-0

**Published:** 2019-10-02

**Authors:** Matthew John Valentine, Courtney Cuin Murdock, Patrick John Kelly

**Affiliations:** 1 One Health Centre for Zoonoses and Tropical Veterinary Medicine, Ross University School of Veterinary Medicine, Island Main Road, West Farm, Basseterre, Saint Kitts and Nevis; 2 Center for the Ecology of Infectious Diseases, University of Georgia, Athens, GA USA; 3 Odum School of Ecology, University of Georgia, Athens, GA USA; 4 Department of Infectious Diseases, University of Georgia, Athens, GA USA; 5 Center for Tropical and Global Emerging Diseases, University of Georgia, Athens, GA USA; 6 Department of Clinical Sciences, Ross University School of Veterinary Medicine, Island Main Road, West Farm, Basseterre, Saint Kitts and Nevis

**Keywords:** Sylvatic, Primates, Enzootic, Arboviruses, Mosquitoes, Chikungunya, Dengue, Zika, Yellow fever

## Abstract

Arboviruses infecting people primarily exist in urban transmission cycles involving urban mosquitoes in densely populated tropical regions. For dengue, chikungunya, Zika and yellow fever viruses, sylvatic (forest) transmission cycles also exist in some regions and involve non-human primates and forest-dwelling mosquitoes. Here we review the investigation methods and available data on sylvatic cycles involving non-human primates and dengue, chikungunya, Zika and yellow fever viruses in Africa, dengue viruses in Asia and yellow fever virus in the Americas. We also present current putative data that Mayaro, o’nyong’nyong, Oropouche, Spondweni and Lumbo viruses exist in sylvatic cycles.

## Background

Many medically important and emergent arboviruses (arthropod-borne viruses) originated in non-human primates (NHPs), which typically show no clinical signs of infection but become viraemic and help maintain the virus in nature [1]. In the natural forest habitats of the NHPs, arboreal mosquitoes transmit arboviruses from infected to naïve animals in what is termed a sylvatic transmission cycle (NHP-mosquito-NHP-mosquito, etc.). People can become infected if they encroach on forest habitat (either through deforestation, hunting, agriculture, or urbanization) and are fed upon by mosquitoes carrying arboviruses, or if infected forest mosquitoes move into areas of human habitation to obtain a blood meal. When humans infected in the forest enter urban environments, arbovirus infections can rapidly spread amongst people transmitted by highly anthrophilic, urban mosquitoes. The sylvatic transmission cycle is then said to have ‘spilled over’ into an urban transmission cycle [2–5]. This is well documented in the case of the yellow fever virus (YFV), where amplifications in infected NHPs precede and lead to short-lived outbreaks in people [3, 6].

Urban transmission cycles of arboviruses in densely populated tropical regions can result in explosive epidemics and pandemics, although there can also be low levels of transmission only sufficient to maintain the viruses in the population. Some arboviruses, like dengue (DENV), chikungunya (CHIKV) and Zika (ZIKV), have become fully adapted to urban cycles and no longer require NHPs, forest mosquitoes and a sylvatic cycle for their maintenance [7]. However, sylvatic cycles could still have important implications for human infections. They may act as refugia for arboviruses which enable re-emergence once human epidemics have passed and immunity in the population (herd immunity) has waned. Further, they might provide selective environments where new strains of arboviruses can develop with increased (or decreased) virulence for people. Also, such novel strains may overcome immunity developed in response to vaccines designed for existing urban strains, so called ‘vaccine redundancy’ [8].

**Fig. 1:**
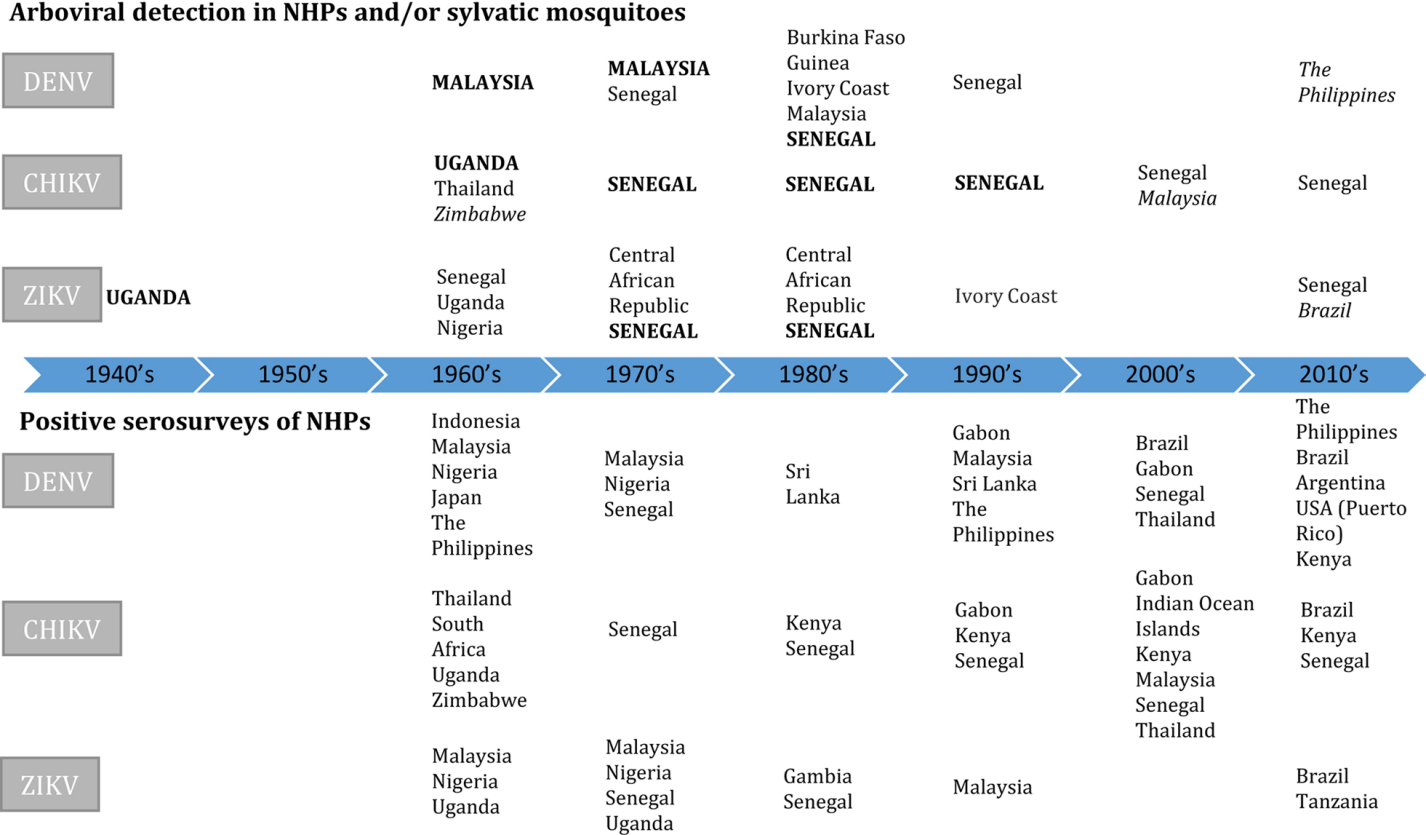
Timeline of investigations of sylvatic cycles in NHPs and sylvatic mosquitoes around the world since the 1950’s. Countries capitalised in bold indicate arboviral isolation in both sylvatic mosquitoes and NHPs. Countries in italics indicate arboviral isolation in NHPs only

Because of the potential significance of sylvatic cycles, leading researchers [3, 9–11] have emphasised the importance of future investigations into their roles in the epidemiology of devastating diseases like dengue, chikungunya, yellow fever and Zika. Below we review the methods used to investigate sylvatic cycles and available epidemiological data on sylvatic cycles of arboviruses in NHPs, primarily focusing on the most recent and largest arbovirus outbreaks in people (Fig. 1).

## Investigation of sylvatic cycles

For the purposes of this review, we regard a sylvatic cycle to be present if there is evidence of biological transmission (i.e. viral isolation, serological testing, blood-meal analysis and experimental infection) of an arbovirus between a NHP (the vertebrate host) and mosquitoes (vectors) that feed on them in a forest ecosystem (Table 1) [3, 7, 10–15].

**Table 1:** Criteria and techniques used to establish the presence of arboviral sylvatic cycles

Criteria	Techniques employed to demonstrate the criteria
Sufficient populations of immuno-naïve (susceptible) NHPs	Antibody detection
Sufficient viraemia in the NHP to infect feeding mosquitoes	Viral isolation from blood
	Viral genome detection from blood
	Antibody detection
Sufficient populations of spatially and temporally coincident competent (capable of viral transmission) infected mosquitoes that feed on NHPs	Mosquito capture and identification
	Virus detection in mosquitoes
	Blood-meal analysis of fed mosquitoes
	Observations of NHPs being fed upon by forest mosquitoes
	Viral transmission experiments (between NHPs and mosquitoes)

### Determining the infection status of the NHP

As stated by Kuno & Chang [15], “The three commonly used data for identifying vertebrate reservoirs for arboviruses have been (i) virus isolation from suspected animals, (ii) relatively high antibody prevalence in the animals captured in the field and (iii) demonstration of viraemia (of higher virus titre and duration) in the suspected animals typically obtained under laboratory conditions” [15].

#### Viral isolation from suspected animals

Isolation of virus from forest NHPs is strong evidence for a sylvatic cycle, however arboviral viremias are only short lived, from one to seven days [16–19]. Thus, finding virus, or identifying fragments of its genome in the case of PCR, in naturally infected animals during field studies is serendipitous [20–24].

#### Relatively high antibody prevalence in the animals captured in the field

Although antibody detection has been used to implicate NHPs in sylvatic cycles in more recent studies [10, 25, 26], stand-alone serology of NHPs, particularly by ELISA, is weak evidence of sylvatic transmission because antibodies only indicate previous exposure (potentially years) of the NHP to the virus and make no assessment of viral kinetics or transmission.

#### Demonstration of viraemia (of higher virus titre and duration) in the suspected animals typically obtained under laboratory conditions

The final evidence for a sylvatic cycle involves the experimental demonstration of a viraemia of sufficient titre to enable mosquitoes to become infected with subsequent transmission to other NHPs [14, 15]. These data are typically obtained under laboratory conditions.

### Determining the infection status of forest-dwelling mosquitoes

There are many different methods that can be used to investigate the mosquito-related criteria for establishing the presence of sylvatic cycles (Table 1). Various sampling techniques have been described to catalogue adult species of mosquito potentially involved in sylvatic cycles in the field [27]. These are based mainly on the target mosquito species and availability of resources and most commonly include, singly or in combination, human landing collections [28], specially designed traps [28, 29], hand-held sweep nets, animal-baited net traps [30] and aspirators [31].

Diallo et al. [28] stated that human landing catches are “the only effective method for sampling sylvatic *Aedes*”; however, humans must be vaccinated against YFV and using malaria prophylaxis. Further, some would consider this technique unethical. There are a variety of mosquito traps that use attractants such as light, carbon dioxide, lures (specially designed scents) or animal bait, but often their use is hindered by limited resources in remote locations. Additionally, traps may require a power source to operate a fan and must be set and checked daily to prevent captured mosquitoes from desiccation which renders them unidentifiable. Animal-baited mosquito net traps are cheap and easy to use but mosquitoes readily escape and the enclosure can alter mosquito behaviour [27]. Handheld sweep nets and aspirators are labour intensive and not species-specific but aspiration is more successful at capturing blood-fed individuals [27]. Hosts on which mosquitoes feed can be discovered by direct observation [23] or by genotyping the blood meal found in engorged mosquitoes, so called blood meal analysis [32, 33].

Demonstration of virus in remote forest-dwelling mosquitoes that cohabit with and feed upon NHPs is considered reliable evidence for a sylvatic cycle [28–30], especially when combined with concurrent serological surveys or viral detection in NHPs. Viral presence can be determined by inoculation of cell cultures with homogenised monospecific pools of 30–50 mosquitoes and identification of virus in the supernatant by IFA, CFT, neutralisation tests or PCR on extracted RNA [28–30].

Confirming the above criteria (Table 1) and establishing with certainty that sylvatic cycles are present is often difficult in practice. Some studies, principally the early ones, established the existence of sylvatic cycles using the above criteria [20–24, 29, 30]. Other studies have relied more on investigating viral presence in either sylvatic mosquitoes [28] or NHPs [10].

### Laboratory tests used to investigate sylvatic cycles

Laboratory methods used to detect arboviral infections in NHPs are virus isolation, nucleic acid amplification by PCR, and serological techniques [34, 35], while detection of infections in mosquitoes depend on virus isolation and/or PCR. As NHPs are only viraemic for relatively short periods after infection, serological testing is frequently used for demonstrating infections that generally result in the production of long-lasting antibody responses [16, 18, 19, 34].

#### Arboviral detection in NHPs and mosquitoes

Virus isolation is the gold standard for identifying infections in NHPs and mosquitoes and has the added advantage of providing viral isolates that can be fully investigated and characterised. It can be performed *in vivo*, originally by inoculation of infant mice, or *in vitro* by cultivation in mosquito cell lines (C6/36 *Aedes albopictus* or AP61 *Aedes pseudoscutellaris*) or mammalian cell lines (e.g. Vero). Virus isolation can be technically challenging, expensive and time consuming and is now only performed in laboratories with appropriate safety facilities [34].

Polymerase chain reaction testing is rapid, sensitive and specific and has now largely replaced viral isolation [34]. However, the sensitivity of the test means it can be prone to false negatives and the specificity depends on the primer design.

#### Antibody detection in NHPs

**Fig. 2:**
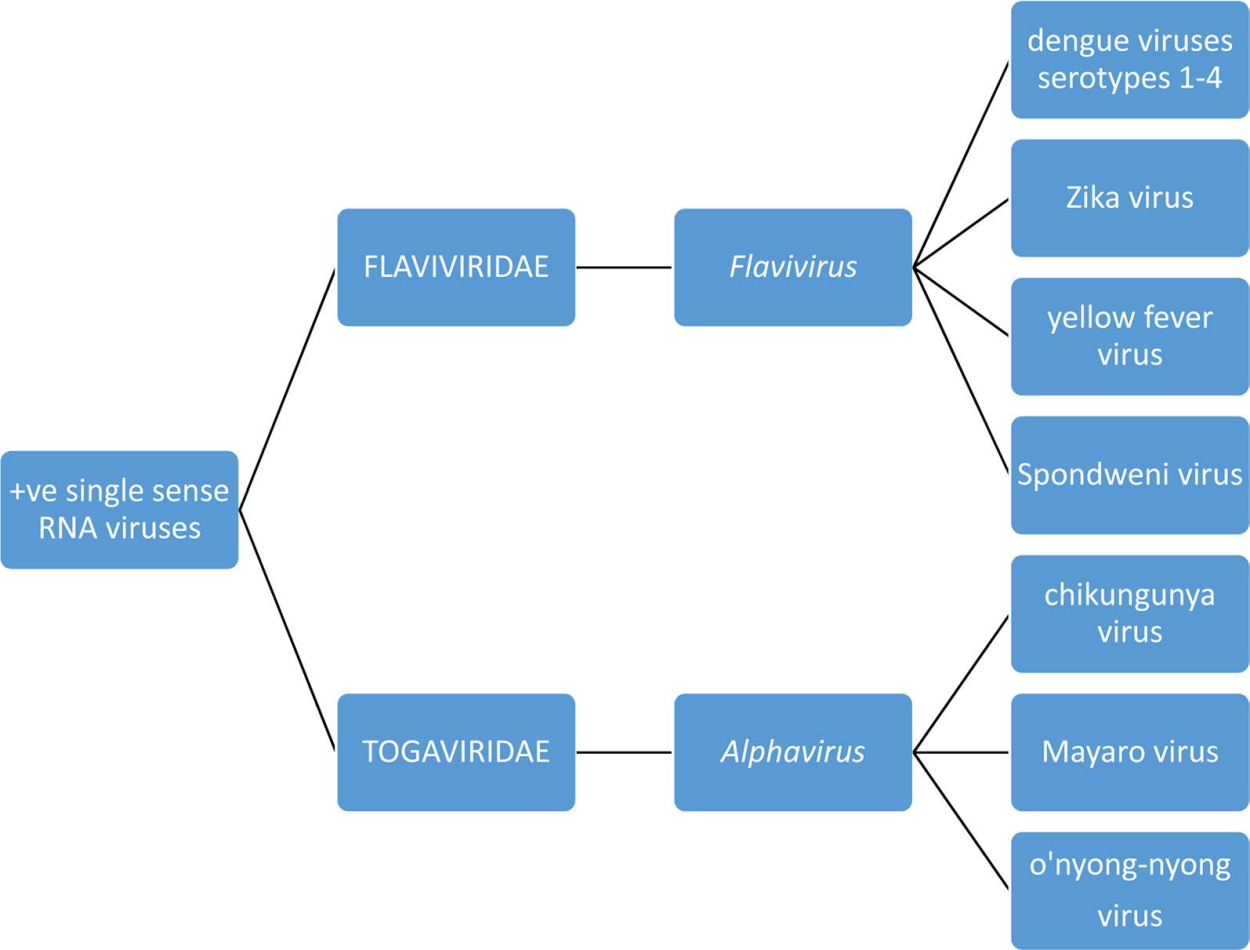
Relationship and classification of arboviruses with known and putative sylvatic cycles involving NHPs

Arboviruses are a diverse group of nearly 500 viruses that are distributed across nine different viral families that share common morphological and molecular characteristics [35, 36]. With regard to sylvatic cycles in NHPs, the DENV, YFV, ZIKV and Spondweni (SPONV) are flaviviruses belonging to the family *Flaviviridae* while CHIKV, Mayaro (MAYV) and o’nyong’nyong (ONNV) are alphaviruses within the *Togaviridae* (Fig. 2). All are single-strand +ve-sense RNA viruses with the members of each family having close antigenic relationships to one another. Unfortunately, these antigenic similarities within the taxa can induce cross-reactive antibody responses causing uncertainty with serological diagnostic tests [34, 35]. Additionally, the concept of original antigenic sin arises when sequential infections by different arboviruses leads to a greater antibody response to the virus responsible for the first infection. This is a particular problem with the multiple serotypes of DENV. Additionally, DENV and ZIKV (*Flaviviridae*) possess similar antigenic surface epitopes (*Flavivirus* E protein) therefore eliciting indistinguishable antibody responses and false positives by some serological techniques [37, 38]. Alphaviruses have similar problems where, for example, methods targeting CHIKV antibodies to the E2EP3 protein (anti-E2EP3) cross-react with non-CHIKV alphaviruses [39] like MAYV or ONNV. Fortuitously, anti-E2EP3 can be used to differentiate between CHIKV infections and *Flavivirus* infections with 93% accuracy [39]. It should be noted that some larger serological studies report results in terms of viral genera, i.e. *Alphavirus-* or *Flavivirus*-positive, thus not distinguishing the species.

#### Haemagglutination inhibition (HAI)

The HAI test is based on the fact that many viruses agglutinate erythrocytes *in vitro*. Antibodies present in test sera prevent this haemagglutination. Although HAI antibodies are long-lasting and the test is of high sensitivity, it lacks specificity [40, 41]. However, it remains useful as a rapid screening test for viruses with common antigenic groups in epidemiological studies [34].

#### Complement fixation test (CFT)

Test sera is added to self-antibody coated sheep erythrocytes with a known concentration of exogenous complement proteins and the antigen of interest. If antibody is present in the test sera, antigen-antibody complexes will form and bind complement preventing erythrocyte lysis. The test is rarely used nowadays for diagnosis as it requires a high level of technical ability and has limited specificity as a stand-alone test [34].

#### Enzyme-linked immunosorbent assay (ELISA)

Many commercial human ELISA kits detecting immunoglobulin G (IgG) [and immunoglobulin M (IgM)] against the various arboviruses are available and have frequently been used for screening NHPs, particularly in dengue vaccine studies. Following a primary infection, IgM appear after 6–14 days and remain elevated for 60–90 days; their presence thus indicates recent exposure [42]. On the other hand, IgG are long-lasting and their presence can be evidence of infections many years previously [43]. In general, the commercial ELISAs are relatively simple, can be performed quickly and can be used for NHPs as the conjugated anti-human sera used in the kits also identify non-human primate antibodies. Although they have high sensitivity and are regarded as reliable screening tools, false positive results are possible due to serological cross-reactivity that occurs between similar arboviruses [34].

#### Indirect immunofluorescence assay (IFA)

Antigen is applied to a microscope slide and test sera overlaid. Reactive antibodies are detected with secondary fluorescent-labelled antisera and fluorescence microscopy. The test is highly sensitive but cross-reactions between viral genera and species reduce the specificity [34].

#### Plaque reduction neutralisation test (PRNT)

This is the gold standard test to demonstrate the presence of specific neutralising antibodies to the various arboviruses. Test sera containing neutralising antibody are added to virus infected cell culture monolayers to inhibit viral plaque formation. Positive results are reported as the dilution that produces a 50% or more reduction in plaque formation. The PRNT is quantitative and specific but unfortunately is time consuming, labour-intensive, and requires a source of live virus and cell culture with appropriate laboratory containment facilities [34].

#### Older neutralisation techniques

In the past, the ability of antibodies in test sera to protect mice against viral challenge has been used to determine seropositivity [44, 45]. As this test requires live animals, it is expensive and contravenes the ‘3 Rs’ (replace, reduce and refine) guiding principles of the use of animals in scientific research [46].

#### Blood-meal analysis by PCR of fed mosquitoes

To determine the host ranges of mosquitoes, DNA can be extracted from the blood meals ingested by engorged females and tested by PCR for vertebrate host species-specific genes, for example the hydroxymethylbilane synthase (HMBS) gene. Sequencing of the PCR amplicons reveals the vertebrate sources of the blood meal with species that have genomic data available [32, 33].

## Natural dengue virus infections in NHPs and mosquitoes

Dengue virus infections are responsible for more deaths and illness than any other arboviral infection in people across tropical and subtropical regions of the world, particularly in Asia and cases have recently increased in Africa [47]. Typical symptoms of infection include fever, myalgia, arthralgia and rashes (dengue fever) or more rarely, a life-threatening haemorrhagic diathesis and circulatory failure (severe dengue or dengue haemorrhagic fever). Dengue virus is a *Flavivirus* (Fig. 2) and exists in four ecologically distinct serotypes.

### Dengue virus infections in NHPs and sylvatic mosquitoes in Asia

Following the elucidation of the ‘jungle cycle’ of yellow fever virus in the early 20th century, the possibility of DENV occurring in a sylvatic cycle in Southeast Asia was mooted by Simmons et al. [48]. During extensive experimental investigations in the 1930’s in the Philippines, they found that NHPs from dengue-free areas could be infected with DENV and mosquitoes that fed on them could subsequently transmit the virus. In contrast, NHPs from dengue-endemic areas were resistant to DENV challenge because they had already been exposed to the virus and developed immunity.

More than 20 years later, Smith [49] postulated that arboreal animals on the Malaysian peninsula were a reservoir for DENV and that infections were maintained by mosquitoes that do not feed at ground level. Hammon et al. [50] reported neutralising antibodies in urban monkeys in Bangkok, but they did not test for reactivity with arboviruses closely related to DENV and, although the specificity of the results were questioned, the possibility of ‘jungle dengue’ in Malaysia was not excluded [51].

Conclusive proof of a sylvatic cycle of DENV involving NHPs in the forests of Malaysia was provided by an extensive study between 1962 and 1980 involving domestic animals (cats, chickens, cattle, dogs, ducks, geese, horse and pigs), over 8000 wild vertebrates (mudskippers, amphibians, reptiles, rodents, birds, insectivores and bats) and over 700 NHPs (Table 2) [23, 52]. After an initial survey from 1962 to 1964 (Table 2), substantial numbers of NHPs were found to have been exposed to DENV, being seropositive by HAI and/or PRNT and with seroconversion demonstrated in some cases (Table 2). While other vertebrate species were positive for antibodies by HAI, their sera failed to neutralise DENV indicating the presence of cross-reacting antibodies against, for example, Japanese encephalitis virus (JEV). Attempts to isolate DENV from mosquitoes, animal sera and tissues (liver, lung, spleen and heart) by inoculation into infant mice were initially only successful with a single NHP in 1968, representing the first recovery of DENV from a vertebrate other than man and later in 1972 from three sentinel dusky leafed monkeys (*Presbytis obscura*) (Table 2).

**Table 2:** Chronological arrangement of reports of laboratory confirmed natural infections of NHPs with DENVs in countries around the world

Year	Reference	Country	Primate species^a^	Diagnostic test	Prevalence (%)
1962–1964	Rudnick (1986) [23]	Malaysia	Silvered leaf monkey (*Presbytis cristatus*)	HAI and SN	66.7 (26/39) and 47.8 (30/63)
1962–1964	Rudnick (1986) [23]	Malaysia	Crab-eating macaque (cynomolgus monkey) (*Macaca fascicularis*)	HAI and SN	92.8 (64/69) and 68.7 (106/154)
1962–1964	Rudnick (1986) [23]	Malaysia	Southern pig-tailed macaque (*Macaca nemestrina*)	HAI and SN	50.0 (1/2) and 50.0 (1/2)
1962–1964	Rudnick (1986) [23]	Malaysia	Sumatran surili (*Presbytis melalophos*)	HAI and SN	75.0 (3/4) and 100 (2/2)
1963–1966 and 1978	Yuwono et al. (1984) [53]	Japan (imported from Indonesia and the Philippines)	Southeast Asian cynomolgus (*Macaca iris*)	PRNT	35.8 (145/358)
1968	Rudnick (1986) [23]	Malaysia	Unspecified	VI	100 (1/1)
1969, 1971–1972	Fagbami et al. (1977) [63]	Nigeria	Unspecified	HAI and SN	48.9 (45/92)
1969, 1971–1972	Fagbami et al. (1977) [63]	Nigeria	Galagos	HAI and SN	25.0 (3/12)
1972	Rudnick (1986) [23]	Malaysia	Dusky leaf monkey (*Presbytis obscura*)	VI	100 (3/3)
1974–1982	Saluzzo et al. (1986) [61]	Senegal	*Erythrocebus patas*; *Cercopithecus aethiops*; *Papio papio*	CFT	2.5 (10/395)
1982	Cornet et al. (1984) [20]	Senegal	Patas monkey (*Erythrocebus patas*)	VI	0.4 (1/250)
1986	De Silva et al. (1999) [57]	Sri Lanka	Toque macaque (*Macaca sinica*)	ELISA	12.5 (2/16)
1987	De Silva et al. (1999) [57]	Sri Lanka	Toque macaque (*Macaca sinica*)	ELISA	93.2 (41/44)
1987	Peiris et al. (1993) [56]	Sri Lanka	Toque macaque (*Macaca sinica*)	PRNT	94.1 (64/68)
1991–2009	Kading et al. (2013) [26]	Gabon	Mandrill (*Mandrillus sphinx*)	PRNT	8.0 (2/25)
1995	De Silva et al. (1999) [57]	Sri Lanka	Toque macaque (*Macaca sinica*)	ELISA	21.3 (52/244)
1996–1997	Wolfe et al. (2001) [58]	Malaysia (Borneo)	Orangutan (*Pongo pygmaeus*)	PRNT	29.6 (21/71)
1999	Inoue et al. (2003) [59]	The Philippines	Cynomolgus (*Macaca fascicularis)*	ELISA	3.7 (2/54)
2000	Diallo et al. (2003) [29]	Senegal	African green monkey (*Chlorocebus sabaeus*)	ELISA	58.8 (10/17)
2006–2014	Catenacci et al. (2018) [67]	Brazil	Golden-headed lion tamarin (*Leontopithecus chrysomelas*)	PRNT	7.7 (8/103)
2008–2009	Nakgoi et al. (2014) [55]	Thailand	Pig-tailed macaque (*Macaca nemestrina leonine*)	PRNT	32.7 (9/38)
2010	Morales et al. (2017) [66]	Argentina	Howler monkey (*Alouatta caraya*)	PRNT	1.8 (2/108)
2010	Kato et al. (2013) [42]	The Philippines	Cynomolgus (*Macaca fascicularis*)	ELISA; PRNT; and PCR	33.0 (33/100); 22.9 (8/35); and 42.8 (3/7)
2010, 2012	Hemme et al. (2016) [65]	USA (Puerto Rico)	Patas monkey (*Erythrocebus patas*)	PRNT	100 (21/21)
2010, 2012	Hemme et al. (2016) [65]	USA (Puerto Rico)	Rhesus macaque (*Macaca mulatta*)	PRNT	100 (2/2)
2013	Moreiro-Soto et al. (2018) [77]	Brazil	Red-handed howler monkey (*Alouatta belzebul*)	PRNT	100 (1/1)
2014	Eastwood et al. (2017) [25]	Kenya	Olive baboon (*Papio anubis*)	ELISA	57.1 (4/7)
2014	Eastwood et al. (2017) [25]	Kenya	Yellow baboon (*Papio cynocephalus*)	ELISA	82.3 (14/17)
2014	Moreiro-Soto et al. (2018) [77]	Brazil	Red-handed howler monkey (*Alouatta belzebul*)	PRNT	100 (1/1)

^a^As listed by the authors

*Abbreviations*: CFT, complement fixation test; ELISA, enzyme-linked immunosorbent assay; HAI, haemagglutination inhibition; PRNT, plaque reduction neutralisation testing; PCR, polymerase chain reaction; SN, serum neutralization; VI, viral isolation

Experimental infections showed that NHPs could be infected with DENV although these animals showed no overt clinical signs. An immune-naïve, wild-caught NHP experimentally infected with a wild type DENV-4 (previously isolated from a NHP) developed DENV-4-specific neutralising antibodies although isolation attempts failed to demonstrate viraemia. Also, a NHP with neutralising antibodies against DENV-1, 2 and 3 became viraemic when infected with the wild type DENV-4 and produced antibodies *de novo* against DENV-4 and substantially increased antibodies against DENV-1, 2 and 3 indicating that although DENV-4 elicits a broadly reactive anti-DENV-1, 2 and 3 antibody response they are not cross-protective to DENV-4. Interestingly, four lorises (*Nycticebus coucang*) were refractory to wild type DENV-4 infection and did not develop antibodies or viraemia indicating that not all NHPs are equally susceptible to DENV.

Rudnick’s studies [23] also included a comprehensive invertebrate survey, primarily targeting mosquitoes to determine their role in sylvatic cycles. Well over 500,000 mosquitoes (in excess of 69 species of 8 genera), representing the majority of species in Malaysia at that time, were collected from tree platforms in the canopy and at ground level using human landing collections, a variety of animal and CO_2_-baited traps, net traps and aspirators. DENV-2 and 4 were isolated from ground level *Ae. albopictus* and DENV-4 from a group of six species of canopy-dwelling mosquitoes reported as *Aedes niveus.* The latter preferentially fed on NHPs when NHP-baited mosquito traps were placed in the forest canopy indicating the species’ probable role in the sylvatic cycle. Ultimately, the study showed there was a sylvatic cycle in the forest canopy in the Malaysian peninsula with all four DENV serotypes circulating between NHPs and mosquitoes (most likely those in the *Ae. niveus* group).

### Dengue virus infections in NHPs in Southeast Asia

Later serosurveys in Southeast Asia described seropositivity in NHPs from Indonesia, the Philippines, Cambodia, Vietnam and Malaysia [53], but not in NHPs originating from Japan (Table 2). Although mosquitoes were not studied, the data were regarded as suggesting sylvatic cycles might be widespread in Southeast Asia. Later, IgG, IgM, neutralising antibody and DENV RNA were demonstrated in cynomolgus macaques (*Macaca fascicularis*) in the Philippines, further suggesting natural cycling of infection in the region (Table 2) [42]. However, sequence analysis of the genes coding for the DENV non-structural protein (NS1) and envelope (E) in isolates from two of the macaques showed they were the same as the circulating human/urban DENV strain. Infections were, then, most likely part of the urban cycle (‘spillback’ or ‘reverse zoonosis’) with NHPs having the potential to act as a reservoir of infection for epidemic/urban strains of the virus. The reverse event, ‘spillover’, was demonstrated to be possible in 2008 in Malaysia when the virus isolated from a man who contracted severe dengue after visiting Rudnick’s field sites grouped into the same clade as Rudnick’s original NHP isolates from 40 years previously [54]. Seropositive NHPs have been found in Thailand suggesting sylvatic cycles might occur there also (Table 2) [55] although there have been no sylvatic mosquito or viral characterisation studies.

There is now evidence that sylvatic cycles in Southeast Asia also occur on islands within the region. In Sri Lanka, almost all the NHPs studied in Polonnaruwa in 1987 had neutralising antibody to DENV-2 (Table 2) [56]. Further analysis revealed a highly focal epizootic of DENV had occurred in the population (Table 2) [57], which was not associated with a concurrent human outbreak. On the island of Borneo (Malaysia), nearly a third of wild and semi-captive orangutans (*Pongo pygmaeus*) sampled were seropositive to DENV-2 (Table 2) [58] and a few captive cynomolgus (*Macaca fascicularis*) on the nearby Luzon Island in the Philippines were IgM positive to DENV and all of them were IgG positive by ELISA against one or more flaviviruses (JEV and/or DENV) (Table 2) [59] suggesting a subacute primary infection or a reinfection.

### Dengue virus infections in NHPs and sylvatic mosquitoes in Africa

Early evidence of a DENV sylvatic cycle in Africa can be found in a review of French publications resulting from work at the Institut Français de Recherche Scientifique pour le Développement en Coopération between 1972 and 1982 [60]. They reported isolating DENV-2 from a forest-dwelling mosquito, *Aedes luteocephalus*, in eastern Senegal in 1972 and because this mosquito was discovered far from human habitation, it was suggested that there might also be a sylvatic cycle of DENV in Africa.

In 1982, DENV-2 was isolated from a patas monkey (*Erythrocebus patas*) (Table 2) [20] in eastern Senegal. Further serological investigations (Table 2) [61] indicated there had been an amplification of DENV infections in local NHPs without a concurrent outbreak in the human population. Also, 28 viral isolations were made from forest-dwelling *Ae. luteocephalus*, *Aedes taylori*-*furcifer*, *Aedes opok* and *Aedes africanus* in the Ivory Coast [60] although there was no evidence of disease in the human population. Similar findings in forest-dwelling *Aedes* spp. in Burkina Faso and Guinea at around the same time added further evidence that these mosquitoes may be involved in a sylvatic cycle of DENV [60]. Ultimately, Rodhain [60] concluded that the studies he reviewed indicated a sylvatic cycle existed in West Africa comprising the patas monkey (*E. patas*) and most likely *Ae. luteocephalus*. The levels of infection varied with amplifications being seasonal, especially in times of high rainfall. However, ‘spillover’ into local human populations appeared uncommon with only one report of DENV-2 being isolated from local people and the sylvatic mosquitoes *Ae. furcifer*, *Ae. taylori* and *Ae. luteocephalus* in 1990 during a dengue epidemic [62].

Further support for the presence of sylvatic cycles in West Africa came from studies in remote forests of Nigeria where Fagbami et al. [63] (Table 2) reported high proportions of NHPs were seropositive against DENV. However, many were simultaneously positive against other flaviviruses indicating either co-circulation of multiple flaviviruses or broadly reactive antibody responses.

Following the initial isolation of DENV-2 from mosquitoes and NHPs in Kedougou, south-eastern Senegal, there have been ongoing comprehensive investigations of sylvatic arbovirus transmission cycles in the forests and savannah of the region [8, 12, 29]. A variety of seropositive NHPs have been reported (Table 2) and further DENV-2 isolates made in cell cultures (AP61; *Aedes pseudoscuttelaris*) from mosquitoes collected in the forest gallery, mainly *Ae. furcifer*, *Ae. taylori*, *Ae. luteocephalus*, *Ae. aegypti* and *Ae. vittatus*. The presence of infected mosquitoes and NHPs was regarded as evidence of a sylvatic cycle of DENV-2 existing in Senegal which involved AGMs in particular [29].

### Dengue virus infections in NHPs in Africa

The recent findings of seropositive NHPs in Kenya (Table 2) [25] adds to the data on seropositive NHPs described in Senegal (Table 2) [29], Nigeria (Table 2) [63] and Gabon (Table 2) [26] and suggests the presence of sylvatic cycles is widespread in Africa.

### Dengue virus infections in NHPs in the Americas

In the Americas, Rosen [64] found none of 105 NHPs sampled during an epidemic of dengue in people in Panama in 1941–1942 were seropositive against DENV, indicating a sylvatic dengue cycle was unlikely to be present at the time. Subsequently, between 2010 and 2012, wild-caught patas monkeys (*E. patas*) and rhesus macaques (*Macaca mulatta*) from Puerto Rico had serological evidence of prior DENV infection (Table 2) [65]. As there was no evidence for sylvatic cycling of DENVs in NHPs in the Americas, the authors suggested their results might represent ‘spillback’ infection, with the NHPs acquiring infections as part of urban cycles involving people on the islands. As viruses were not isolated, it was not possible to use phylogenetic characterisation to confirm the (urban) origin of the viruses.

In a survey in Argentina in 2010 (Table 2) [66], only a few free-ranging howler monkeys (*Alouatta caraya*) were seropositive solely to DENV-1 and 3. This low level of infection was thought to result from ‘spillback’ from human infections rather than being evidence of a sylvatic cycle. Many other NHPs in the study had low magnitude seropositivity to DENV-1 and 3, which was thought to be due to multiple closely related flaviviruses infecting NHPs in a sylvatic environment. In the Bahia Atlantic Forest Reserve of Brazil between 2006 and 2014, low seropositivity to DENVs was found in free-living golden-headed lion tamarins (*Leontopithecus chrysomelas*) that were proximate to agricultural workers thereby raising the possibility of ‘spillback infection’ (Table 2) [67].

### Dengue virus infections in sylvatic mosquitoes in the Americas

There is little evidence of sylvatic cycles from mosquito studies with only a single putative isolation of DENV-1, not molecularly confirmed, from a rainforest-dwelling mosquito, *Haemagogus leucocelaenus*, near Brazil in 2002 [68].

### Summary

Sylvatic cycles of DENVs involving NHPs have been shown to exist in Asia and Africa (Malaysia and Senegal), where DENV has been isolated from forest mosquitoes that feed on NHPs and from NHPs themselves. Serological data imply that sylvatic cycles may exist in the larger environs and islands of Southeast Asia and widely across Africa. There is no good evidence yet for sylvatic DENV circulation in NHPs in the Americas.

## Natural chikungunya virus infection in NHPs and mosquitoes

Prior to 2004, the CHIKV was only known to produce localised outbreaks of disease (chikungunya fever) in Africa and Southeast Asia. Subsequently, there has been a near global spread of CHIKV to La Réunion, India, Asia and Europe reaching the Americas in December 2013, infecting millions of people [47]. Typical symptoms of infection include fever, myalgia, arthralgia, headaches and rashes and chronic arthritis. Rarely, more severe manifestations can include neurological disease, myocarditis and death. The CHIKV is an *Alphavirus* (Fig. 2), which occurs as three different genotypes [47].

### Chikungunya virus infections in NHPs and sylvatic mosquitoes in Africa

McIntosh et al. [22] investigated possible sylvatic cycles of CHIKV in 1962 in Rhodesia (Zimbabwe) and discovered all the NHPs sampled were seropositive, although mosquitoes collected as part of the study, notably including the primatophilic *Ae. furcifer*-*taylori*, failed to yield virus.

In a wider study testing greater numbers of NHPs in South-West Africa (Namibia), South Africa (Cape Province, Orange Free State, Natal, Transvaal), Botswana, Zimbabwe (as Rhodesia) and Mozambique from 1964 to 1969, the same species of NHP were also seropositive (Table 3) [69]. Analysis of the ages of the seropositive AGMs in Natal suggested a recent epizootic although none of the 42,000 mosquitoes tested contained CHIKV and no sentinel NHPs seroconverted [69]. The study indicated there was no active infection and NHPs were not currently maintaining sylvatic CHIKV although other arboviruses could be isolated from mosquitoes suggesting sylvatic transmission was viable in the region at that time [69]. In the forest canopies of Uganda (including Zika forest) in 1969, CHIKV was isolated from sylvatic mosquitoes *Aedes africanus* (9/102 pools; 8.8%) and *Mansonia* (*Mansonoides*) *fuscopennata* (1/97 pools; 1.0%) accompanying high seropositivity in red-tailed monkeys (*Cercopithecus ascanius schmidti*) (Table 3) [70], consistent with an epizootic and probable sylvatic transmission.

**Table 3:** Chronological arrangement of reports of laboratory confirmed natural infections of NHPs with CHIKV in countries around the world

Year	Reference	Country	Primate species^a^	Diagnostic test	Prevalence (%)
1962	McIntosh et al. (1964) [22]	Zimbabwe^b^	Chacma baboon (*Papio ursinis*)	HAI and VI	100 (9/9)
1962	McIntosh et al. (1964) [22]	Zimbabwe^b^	African green monkey (*Chlorocebus aethiops sabaeus*)	HAI and VI	100 (4/4)
1965?	Halstead & Udomsakdi, (1966) [73]	Thailand	Unspecified	HAI and SN	Seropositive, numbers not stated
1963–1967	Marchette et al. (1978) [74]	Thailand	*Macaca fascicularis*	HAI and PRNT	1.5 (6/393)
1963–1967	Marchette et al. (1978) [74]	Thailand	*Macaca nemestrina*	HAI and PRNT	2.3 (3/132)
1963–1967	Marchette et al. (1978) [74]	Thailand	*Presbytis melalophos*	HAI and PRNT	16.7 (1/6)
1963–1967	Marchette et al. (1978) [74]	Thailand	*Presbytis obscura*	HAI and PRNT	14.6 (6/41)
1964–1969	McIntosh (1970) [69]	South Africa (Transvaal)	Chacma baboon (*Papio ursinis*)	HAI and SN	11.9 (9/76)
1964–1969	McIntosh (1970) [69]	South Africa (Natal)	African green monkey (*Chlorocebus aethiops sabaeus*)	HAI and SN	25.1 (75/298)
1964–1969	McIntosh (1970) [69]	Zimbabwe^b^	Chacma baboon (*Papio ursinis*)	HAI and SN	100 (4/4)
1969	McCrae et al. (1971) [70]	Uganda	Red-tailed monkey (*Cercopithecus ascanius schmidti*)	HAI	83.3 (25/30)
1972	Diallo et al. (1999) [30]	Senegal	African green monkey (*Cercopithecus aethiops*)	VI	2/not stated
1975	Diallo et al. (1999) [30]	Senegal	Baboon (*Papio papio*)	VI	1/not stated
1983	Diallo et al. (1999) [30]	Senegal	Patas monkey (*Erythrocebus patas*)	VI	1/not stated
1985–2000	Eastwood et al. (2017) [25]	Kenya	Olive baboon (*Papio anubis*)	PRNT	13.1 (33/252)^c^
1985–2000	Eastwood et al. (2017) [25]	Kenya	Vervet monkey (*Chlorocebus aethiops*)	PRNT	13.1 (33/252)^c^
1985–2000	Eastwood et al. (2017) [25]	Kenya	Blue monkey (*Cercopithecus mitis*)	PRNT	13.1 (33/252)^c^
1996	Diallo et al. (1999) [30]	Senegal	Bush baby (*Galago senegalensis*)	VI	1/not stated
1996	Diallo et al. (1999) [30]	Senegal	African green monkey (*Cercopithecus aethiops*)	VI	1/not stated
1998-2006	Kading et al. (2013) [26]	Gabon	Mandrill (*Mandrillus sphinx*)	PRNT	20.0 (5/25)
2006	Vourcʼh et al. (2014) [72]	Indian Ocean Islands (Réunion, Mayotte, Mauritius)	Brown lemur (*Eulemur fulvus*)	ELISA and PRNT	3.8 (2/52)
2006	Vourcʼh et al. (2014) [72]	Indian Ocean Islands (Réunion, Mayotte, Mauritius)	Crab-eating macaque (*Macaca fascicularis*)	ELISA and PRNT	1.5 (2/134)
2007–2008	Apandi et al. (2009) [75]	Malaysia	Long-tailed macaque (*Macaca fascicularis*)	PCR	3.8 (4/105)
2008–2009	Nakgoi et al. (2014) [55]	Thailand	Northern pig-tailed macaque (*Macaca nemestrina leonine*)	PRNT	10.5 (4/38)
2009–2010	Sam et al. (2015) [76]	Malaysia	Long-tailed macaque (*Macaca fascicularis*)	PRNT	0.7 (1/146)
2009–2010	Sow et al. (2018) [71]	Senegal	Guinea baboon (*Papio papio*)	PRNT	82.0 (96/117)
2009–2010	Sow et al. (2018) [71]	Senegal	African green monkey (*Chlorocebus sabaeus*)	PRNT	75.8 (25/33)
2009–2010	Sow et al. (2018) [71]	Senegal	Patas monkey (*Erythrocebus patas*)	PRNT	7.0 (5/71)
2010–2012	Althouse et al. (2018) [10]	Senegal	Guinea baboon (*Papio papio*)	PRNT	71.8 (479/667) (*n* = 399)^c^
2010–2012	Althouse et al. (2018) [10]	Senegal	African green monkey (*Chlorocebus sabaeus*)	PRNT	71.8 (479/667) (*n* = 198)^c^
2010–2012	Althouse et al. (2018) [10]	Senegal	Patas monkey (*Erythrocebus patas*)	PRNT	71.8 (479/667) (*n* = 70)^c^
2013	Moreiro-Soto et al. (2018) [77]	Brazil	White-cheeked spider monkey (*Ateles marginatus*)	PRNT	33.3 (1/3)
2013	Moreiro-Soto et al. (2018) [77]	Brazil	Common marmoset (*Callithrix jacchus*)	PRNT	12.5 (1/8)
2013	Moreiro-Soto et al. (2018) [77]	Brazil	Capuchin monkey (*Sapajus* sp.)	PRNT	5.4 (2/37)
2013	Moreiro-Soto et al. (2018) [77]	Brazil	Buff-headed capuchin (*Sapajus xanthosternos*)	PRNT	18.2 (2/11)
2014	Eastwood et al. (2017) [25]	Kenya	Olive baboon (*Papio anubis*)	PRNT	27.8 (5/18)
2014	Eastwood et al. (2017) [25]	Kenya	Red-tailed monkey (*Cercopithecus ascanius*)	PRNT	14.3 (1/7)
2014	Eastwood et al. (2017) [25]	Kenya	Blue monkey (*Cercopithecus mitis*)	PRNT	37.5 (3/8)
2014	Moreiro-Soto et al. (2018) [77]	Brazil	Blonde capuchin (*Sapajus flavius*)	PRNT	4.8 (1/21)
2016	Moreiro-Soto et al. (2018) [77]	Brazil	Crested capuchin (*Sapajus robustus*)	PRNT	50.0 (1/2)
2016	Moreiro-Soto et al. (2018) [77]	Brazil	Capuchin monkey (*Sapajus* sp.)	PRNT	14.3 (3/21)

^a^As listed by the authors

^b^As Southern Rhodesia

^c^Distribution of results between NHP species not reported

*Abbreviations*: ELISA, enzyme-linked immunosorbent assay; HAI, haemagglutination inhibition; PRNT, plaque reduction neutralisation testing; PCR, polymerase chain reaction; SN, serum neutralization; VI, viral isolation

In Senegal, between 1972 and 1983, CHIKV was isolated from multiple species of NHP (Table 2) [30]. Over the same period, CHIKV was detected in forest-dwelling mosquitoes using multiple tests. Between 1972 and 1986, Diallo et al. [30] isolated 178 strains of CHIKV, in cell cultures or mice, from 599,582 forest canopy mosquitoes, mainly *Ae. furcifer*-*taylori* (129/8244 pools; 1.6%), *Ae. luteocephalus* (27/3347 pools; 0.8%) and *Ae. dalzieli* (12/2069 pools; 0.6%). A more detailed survey between 2009 and 2010 in the area (now considered a known focus of sylvatic arbovirus circulation due to previous CHIKV isolation in mosquitoes and NHPs) identified CHIKV by multiple tests in 15/50 species of mosquitoes captured [28] with 42 out of 4211 (10%) pools of mosquitoes sampled (39,799 mosquitoes) were positive for CHIKV. Of these positive pools, 16 were *Ae. furcifer* (0.4%), five were *Ae. taylori* (0.1%), and five were *Ae. luteocephalus* (0.1%). It was concluded that *Ae. africanus*, *Ae. luteocephalus* and *Ae. furcifer-taylori* (now recognised as two species: *Ae. furcifer* and *Ae. taylori*) were involved in the maintenance of the sylvatic cycle of CHIKV with *Ae. furcifer* most likely to contribute to any ‘spillover’ of infection to people. Shortly thereafter, when blood meals of the above species were analysed by PCR, only *Ae. taylori* was found to have fed on NHPs [32] indicating that blood meal host preferences vary between mosquito species and that species other than NHPs may be involved.

### Chikungunya virus infections in NHPs in Africa

High seroprevalences have been recorded in multiple species of NHPs in Senegal, which have sometimes been associated with outbreaks in people (Table 3) [10, 71]. Of note, immediately after an outbreak of CHIKV in people in the region from 2010 to 2012 [10], high seropositivities were found in NHPs, especially juveniles. This was considered to indicate the presence of high herd immunity and that ongoing CHIKV circulation in a sylvatic cycle would be unlikely. There was thus the possibility that NHPs were not the only vertebrate hosts involved and alternate reservoirs of CHIKV were possible [10].

In Africa, then, there is strong evidence suggesting sylvatic cycles might occur in several species of NHPs across the continent. There is widespread evidence of exposure to CHIKV in a variety of NHPs across the continent in South Africa and Zimbabwe (Table 3) [22, 69], Uganda (Table 3) [70], Gabon (Table 3) [26] and Kenya (Table 3) [25], and CHIKV has been isolated from multiple species of NHPs [22, 30].

Off the coast of Africa, during the 2006 chikungunya epidemic on the French islands of La Réunion, Mayotte and Mauritius, approximately 266,000 people out of a total of 785,000 individuals were infected in La Réunion alone, but there was little serological evidence of infections in NHPs with only a few brown lemurs (*Eulemur fulvus*) and crab-eating macaques (*Macaca fascicularis*) found seropositive (Table 3) [72]. The brown lemurs (*E. fulvus*) were negative for CHIKV by PCR and it was thought the seropositive animals resulted from ‘spillback’ infections from people rather than as a result of the presence of a sylvatic cycle.

### Chikungunya virus infections in NHPs and sylvatic mosquitoes in Asia

There are sparse data on sylvatic CHIKV in Asia. In rural Thailand in the 1960’s, CHIKV was first isolated from a *Culex tritaeniorhynchus* and antibodies were reported in NHPs (Table 3) [73].

### Chikungunya virus infections in NHPs in Asia

Further serological investigations of 1880 potential vertebrate hosts on the Malaysian peninsula between 1963 and 1967 included 642 NHPs, and only 16 (2.5%) had antibodies to CHIKV (Table 3) [74]. The first isolation of CHIKV from NHPs in Asia was in Malaysia, from wild long-tailed macaques (*Macaca fascicularis*) sampled from 2007 to 2008; PCR and sequencing revealed the virus was distinct from the strain circulating in people at the time (Table 3) [75]. In a subsequent serosurvey the following year, shortly after a nationwide outbreak in people, the prevalence of neutralising antibodies in wild long-tailed macaques (*M. fascicularis*) was very low (1/146; 0.7%) (Table 3) [76]. However, nearby in northern Thailand at about the same time, a higher percentage (4/38; 10.5%) of captive northern pig-tailed macaques (*Macaca nemestrina leonine*) were found to be seropositive before and after CHIKV activity in people in the region suggesting macaques could become infected by CHIKV independently of people (Table 3) [55]. Finally, on the Island of Borneo (Malaysia) wild and semi-captive orangutans (*Pongo pygmaeus*) were seronegative to CHIKV [58] indicating that sylvatic CHIKV may not infect this species or the virus might not have spread to islands in the region.

### Chikungunya virus infections in NHPs in the Americas

The CHIKV was introduced into the Americas in 2013 and 2014 and as the epidemic progressed, it was speculated that there was a risk of the virus establishing a sylvatic cycle in New World NHPs, as YFV had done previously. Investigations of sylvatic cycles have been limited and confined to Brazil [77] where several species of NHP were sampled between 2012 and 2017 and found to have very low seropositivity to CHIKV with very low antibody titres (Table 3) [77]. Two of the NHPs were also MAYV positive by PRNT highlighting the difficulty in interpreting serology in the presence of cross-reactivity. None had detectable CHIKV RNA by RT-PCR indicating an absence of active infection [77].

### Summary

In Africa (Uganda and Senegal), CHIKV has been isolated from forest mosquitoes and NHPs and seropositive NHPs are common and widespread, providing good evidence for the presence of sylvatic transmission cycles. However, it is unclear if NHPs are the only vertebrate host of CHIKV or if there is a role for other vertebrates in the maintenance of the virus. In Asia, a sylvatic cycle has not been demonstrated as there is no evidence of CHIKV in forest-dwelling mosquitoes and CHIKV infections are not common or widespread in NHPs. Similarly, in the Americas, although investigations have been limited, there is only weak serological data suggesting sylvatic cycles, although it has been suggested they might yet become established due to the high numbers of susceptible mosquitoes, NHPs and people. [77–80].

## Natural infections of Zika virus in NHPs and mosquitoes

The ZIKV is endemic in Africa and Asia [47] but caused a global epidemic when it spread to Yap Island in Micronesia in 2007 and eventually through the Pacific islands to the Americas by 2015. Infections in people can be asymptomatic or result in mild fever, rash, arthritis, arthralgia and myalgia. Infections have been linked with Guillain-Barré syndrome and severe birth defects in babies born to women infected while pregnant. The ZIKV is a *Flavivirus* (Fig. 2) and there are three distinct genotypes [47].

### Zika virus infections in NHPs and sylvatic mosquitoes in Africa

The ZIKV was first identified in 1947 in the Zika (syn. Ziika) forest of Uganda (Table 4) [21] when, as part of a YFV surveillance project, the ZIKV was isolated from a sentinel rhesus macaque (*Macaca mulatta*) and the local sylvatic YFV vector, *Ae. africanus*. This provided evidence of a sylvatic circulation of the virus in NHPs and mosquitoes that feed on them. More extensive investigations in the region in the late 1960’s found high seropositivity in several NHP species (Table 4) [81] and led to the isolation of ZIKV from *Ae. africanus* (14/27 pools; 51.8%) and *Aedes apicoargenteus* (1/1 pool; 100%) [81]. Due to the extensive arboviral investigations and periodic YFV and CHIKV activity in the NHPs and mosquitoes, researchers began to suggest the possibility of cross-protection (immunisation) between YFV and ZIKV (and possibly CHIKV) in NHPs. It was also suggested that successive arboviral infection (flaviviruses and alphaviruses) of mosquitoes may interfere with their ability to transmit virus [81]. In 1968 in Senegal, the ZIKV was first isolated from *Ae. luteocephalus* from the Saboya forest and in 1980 the ZIKV was also isolated from NHPs (Table 4) [8, 9, 16].

**Table 4:** Chronological arrangement of reports of laboratory confirmed natural infection of NHPs with ZIKV in countries around the world

Year	Reference	Country	Primate species^a^	Diagnostic test	Prevalence (%)
1947	Dick et al. (1952) [21]	Uganda	Rhesus macaque (*Macaca mulatta*)	VI	100 (1/1)
1962–1971	Rudnick (1986) [23]	Malaysia	Pig-tailed macaque (*Macaca nemestrina*)	HAI	1.2 (2/163)
1962–1971	Rudnick (1986) [23]	Malaysia	Long-tailed macaque (*Macaca fascicularis*)	HAI	1.8 (4/225)
1969–1970	McCrae & Kirya (1982) [81]	Uganda	Red-tailed monkey (*Cercopithecus ascanius schmidti*)	HAI and SN	38.1 (54/142) and 52.1 (74/142)
1969–1970	McCrae & Kirya (1982) [81]	Uganda	Colobus (*Colobus abyssinicus uellensis*)	HAI and SN	45.4 (5/11) and 54.5 (6/11)
1969–1970	McCrae & Kirya (1982) [81]	Uganda	Mangabey (*Cercocebus albigena johnstoni*)	HAI and SN	50.0 (2/4) and 75 (3/4)
1969–1971	Monath & Kemp (1973) [45]	Nigeria	African green monkey (*Chlorocebus aethiops*)	HAI and SN	55.5 (5/9) and 66.6 (6/9)
1969–1971	Monath & Kemp (1973) [45]	Nigeria	Mona monkey (*Cercopithecus mona*)	HAI and SN	36.1 (13/36) and 41.7 (15/36)
1969–1971	Monath & Kemp (1973) [45]	Nigeria	Western putty-nosed monkey (*Cercopithecus nictitans martini*)	HAI and SN	50.0 (2/4) and 25.0 (1/4)
1969–1971	Monath & Kemp (1973) [45]	Nigeria	Red-capped mangabey (*Cercopithecus torquatus*)	HAI and SN	100 (5/5) and 80.0 (4/5)
1969–1971	Monath & Kemp (1973) [45]	Nigeria	Olive baboon (*Papio anubis choras*)	HAI and SN	100 (2/2) and 50.0 (1/2)
1969–1971	Monath & Kemp (1973) [45]	Nigeria	Wadi monkey (*Erythrocebus patas*)	HAI and SN	11.9 (8/67) and 5.9 (4/67)
1979–1980	Althouse et al. (2015) [8]	Senegal	Vervet monkey (*Chlorocebus aethiops*)	VI and IFA	100 (1/1) and 100 (1/1)
1980	Althouse et al. (2015) [8]	Senegal	Patas monkey (*Erythrocebus patas*)	VI and IFA	100 (1/1) and 100 (1/1)
1985, 1986	Buechler et al. (2016) [84]	Gambia	Vervet monkey (*Chlorocebus* sp.)	ELISA	16.0 (4/25)
1996–1998	Kilbourn et al. (2003) [91]	Malaysia	Western Bornean orangutan (*Pongo pygmaeus pygmaeus*)	ELISA and/or IFA	8.4 (6/71)
1996–1997	Wolfe et al. (2001) [58]	Malaysia	Western Bornean orangutan (*Pongo pygmaeus pygmaeus*)	PRNT	2.8 (2/71)
2010–2014	Buechler et al. (2016) [84]	Tanzania	Yellow baboon (*Papio* sp.)	ELISA	4.9 (2/41)
2012	Moreiro-Soto et al. (2018) [77]	Brazil	Capuchin (*Sapajus* sp.)	PRNT	4.8 (1/21)
2012	Moreiro-Soto et al. (2018) [77]	Brazil	Marmoset (*Callithrix penicillata*)	PRNT	100 (1/1)
2013	Moreiro-Soto et al. (2018) [77]	Brazil	Red-handed howler monkey (*Alouatta belzebul*)	PRNT	100 (1/1)
2014	Moreiro-Soto et al. (2018) [77]	Brazil	Capuchin (*Sapajus flavius*)	PRNT	4.8 (1/21)
2014	Moreiro-Soto et al. (2018) [77]	Brazil	Red-handed howler monkey (*Alouatta belzebul*)	PRNT	100 (1/1)
2016	Favoretto et al. (2016) [92]	Brazil	Capuchin (*Sapajus libidinosus*)	PCR	33.3 (3/9)
2016	Favoretto et al. (2016) [92]	Brazil	Marmoset (*Callithrix jacchus*)	PCR	26.7 (4/15)
2017	Terzian et al. (2018) [93]	Brazil	Capuchin (*Sapajus libidinosus*)	PCR	38.2 (31/81)
2017	Terzian et al. (2018) [93]	Brazil	Marmoset (*Callithrix jacchus*)	PCR	100 (1/1)
2017	Moreiro-Soto et al. (2018) [77]	Brazil	Red-handed howler monkey (*Alouatta belzebul*)	PRNT	33.3 (1/3)

^a^As listed by the authors

*Abbreviations*: ELISA, enzyme-linked immunosorbent assay; HAI, haemagglutination inhibition; IFA, immunofluorescence antibody test; PRNT, plaque reduction neutralisation testing; PCR, polymerase chain reaction; SN, serum neutralization; VI, viral isolation

### Zika virus infections in sylvatic mosquitoes in Africa

Subsequent investigations of mosquitoes in forests and forest canopies have identified further species naturally infected with ZIKV: *Ae. opok* in the Central African Republic; *Ae. luteocephalus* in Nigeria; and *Ae. vittatus*, *Ae. furcifer* and *Ae. aegypti formosus* in the Ivory Coast and Senegal [82]. It is assumed that because these species were collected in remote forest areas, away from human habitation, transmission of ZIKV is primarily sylvatic involving NHPs, although the possibility of vertical transmission could not be excluded [83].

### Zika virus infections in NHPs in Africa

As the recent ZIKV pandemic developed, serological evidence of natural infections of ZIKV were described in a wide variety of NHPs from across Africa including Uganda, Nigeria, Gambia and Tanzania (Table 4) [45, 47, 84–86].

### Zika virus infections in NHPs and sylvatic mosquitoes in Asia

In Asia, there have been only limited studies of possible ZIKV vectors [87, 88] despite the widespread but benign circulation of ZIKV in people for decades [89]. Marchette et al. [90] investigated potential sylvatic mosquito vectors across Malaysia and despite analysing 27,636 *Aedes* spp. mosquitoes from rural areas, rain forests, mangrove swamps and freshwater swamps, they only managed to isolate ZIKV from urban *Ae. aegypti*. Interestingly, the authors state “there is strong serological evidence that it (ZIKV) occurs naturally in wild monkeys in Malaysia (our unpublished data)”.

### Zika virus infections in NHPs in Asia

The only published data on NHPs revealed ZIKV seropositive individuals in a small focus of captive and semi-wild orangutans (*Pongo pygmaeus*) in Borneo, Malaysia, which was thought to represent ‘spillback’ from infected people (Table 4) [58, 91].

### Zika virus infections in NHPs in the Americas

In the Americas, ‘spillback’ infection from people to peri-urban capuchins (*Sapajus libidinosus*) and marmosets (*Callithrix jacchus*) in two cities in Brazil was suspected to be the reason for several ZIKV RT-PCR-positive NHPs found in 2016 and 2017 during the large human epidemic (Table 4) [92, 93]. This finding rekindled speculation that a sylvatic cycle of ZIKV could become established. More extensive studies around Brazil, however, failed to identify ZIKV RNA by PCR and found only low seroprevalences and antibody titres to ZIKV, which were thought to indicate an absence of established sylvatic cycles (Table 4) [77].

### Summary

Sylvatic cycles of ZIKV exist in Africa (Uganda and Senegal) with virus having been isolated from forest-dwelling mosquitoes and NHPs which appear to be commonly infected based on widespread seropositivity. Sylvatic cycles appear to be absent in Asia but there are limited data for this region. Currently, sylvatic cycles of ZIKV have not been conclusively demonstrated in the Americas; however, multiple examples of ‘spillback’ infection are documented and there is thus a possibility for sylvatic cycles to develop [9, 82].

## Natural yellow fever virus infections in NHPs and mosquitoes

The history and role of NHPs in the epidemiology of YFV around the world is well documented [3, 6, 60, 94] and the early investigations into the epidemiology of YFV led to the development of the concepts of arboviral sylvatic cycles in NHPs that we have today [13, 94]. Yellow fever outbreaks continue in Africa and South America and can be extensive. Most YFV infections in people are subclinical but signs can range from mild fever, myalgia, headache and nausea with a rapid recovery to high fever, abdominal pain, jaundice, hepatitis, haemorrhagic diatheses and death [47]. The YFV is the archetypal *Flavivirus* and exists in seven genotypes [6].

### Yellow fever infection in NHPs and mosquitoes in Africa

YFV originated in Africa in a sylvatic cycle involving a wide range of NHPs including baboons (*Papio* spp.), colobus monkeys (*Colobus* spp.), green and vervet monkeys (*Cercopithecus* spp.), mangabeys (*Cercocebus* spp.), chimpanzees (*Pan troglodytes*), bush babies (*Galago* spp.) and forest-dwelling mosquitoes (*Aedes bromeliae*, *Ae. taylori*, *Ae. africanus*, *Ae. luteocephalus*, *Aedes metallicus*, *Ae. opok*, *Ae. vittatus* and the *Aedes simpsoni* complex) [3, 82].

### Yellow fever infection in NHPs and mosquitoes in the Americas

The YFV was introduced into people in the Americas during the slave trade with West Africa 400 years ago [3]. Sylvatic cycles developed in South America as local NHPs became infected, mainly howler monkeys (*Alouatta* spp.), squirrel monkeys (*Saimiri* spp.), spider monkeys (*Ateles* spp.) and owl monkeys (*Aotus* spp.). Transmission of the YFV in the NHPs was established by infection of local mosquitoes that were capable of transmitting the YFV, mainly *Haemagogus albomaculatus*, *Haemagogus spegazzini*, *Haemagogus janthinomys*, *Sabethes chloropterus*, *Sabethes albipivus*, *Sabethes glaucodaemon*, *Sabethes soperi* and *Sabethes cyaneus* [3, 82].

Unlike the situation with NHPs in Africa, native South American species of NHP are susceptible to YFV, developing clinical signs and often dying from infections. Indeed, today nearly all human outbreaks of YFV in South America and Africa result from ‘spillover’ infection from NHPs, and deaths in South American NHPs are now a well-known warning sign of YFV amplification and the heralding of a yellow fever outbreak in local people [3, 6, 82].

### Yellow fever infection in NHPs and mosquitoes in Asia

Yellow fever is notably absent from Asia despite there being large numbers of susceptible people, mosquitoes that can transmit the virus, and native NHPs that have been found to be highly susceptible to experimental infections with YFV [6]. Possible reasons for the absence of observed disease in people have been reviewed [3] and include the lack of opportunity for the YFV to spread with the lack of major trade routes between West Africa and Asia and the fact that YFV has not been documented in nearby East Africa. It might also result from lack of surveillance and the similar clinical presentation of yellow fever and dengue, which is a very common disease in Asia. Additionally, DENV antibodies in NHP hosts and people might provide cross-protection against the YFV and there might be direct viral competition between the viruses in mosquitoes. There is also the unlikely possibility that Asian *Aedes* spp. cannot transmit YFV.

## Future threats

Several other mosquito-borne viruses that produce disease in people are suspected to be sustained in nature by sylvatic cycles involving NHPs and/or other vertebrates. Presently, they appear localised and to cause little human disease but have the potential for geographical expansion with changes in climate, mosquito distribution, viral genomic drift, and encroachment of people into the sylvatic habitats [3–5, 13, 47]. The putative involvement of NHPs in the sylvatic transmission of these viruses is largely based on retrospective serological investigations with all their limitations.

### Mayaro virus

MAYV (*Alphavirus*) was first discovered in a group of rural workers in Trinidad in 1954 [95] and subsequently reported to occur in a sylvatic cycle in South America, probably involving NHPs and forest mosquitoes (*Haemagogus* spp.). The virus, however, has never been isolated from an NHP and the definitive reservoir host is still unproven with antibodies having been detected in many vertebrates [96, 97]. In Panama, a high prevalence of neutralising antibodies was reported in howler monkeys (*Alouatta villosa*) [97], and in French Guiana, anti-MAYV HAI antibodies have been detected in red howler monkeys (*Alouatta seniculus*) (70/106; 66%) and golden-handed tamarins (*Saguinus midas*) (8/44; 18%) [98]. Subsequently, they were found again in red howler monkeys (*A. seniculus*) and golden-handed tamarins (*S. midas*) (19/42; 50%) in addition to (52/98; 53%), white-faced saki (*Pithecia pithecia*) (5/80; 6%) and squirrel monkeys (*Saimiri sciureus*) (6/67; 9%) [96]. More recent studies in Brazil in 2010 and 2014, have demonstrated anti-MAYV HAI antibody in free-living capuchins (*Sapajus* spp.) [41, 99], black howler monkeys (*Alouatta caraya*) [41] and captive NHPs [black howler monkeys (*Alouatta caraya*), white-eared titis (*Callicebus donacophilus*) and tufted capuchins (*Cebus apella*)] [40]. There may also be alternate or additional non-primate vertebrate hosts, and it has been suggested that MAYV is introduced into canopy-dwelling NHPs by migratory birds [40]. This might account for higher serological evidence of infection in NHPs more active in tree canopies [96].

### Oropouche virus

Oropouche virus (OROV) (*Orthobunyavirus*) causes Oropouche fever in people and has been responsible for large epidemics (second only to dengue) in Brazil, Peru, Panama, and Trinidad and Tobago where it was initially isolated from a forest worker in 1955 [100]. The OROV exists in a sylvatic cycle reportedly involving the mosquito species *Coquillettidia venezuelensis*, *Aedes serratus*, *Culex quinquefasciatus*, *Ochlerotatus serratus* and midges of the genus *Culicoides*. A vertebrate host has not been established, but there is a single report of isolation of OROV from a marmoset in Brazil (*Callithrix* sp.) [101] and a sloth (*Bradypus tridactylus*) in a 1960 report. A few free-living and captive NHPs in Brazil have been found seropositive [40, 41], as have capuchin monkeys (*Sapajus* spp.), black-and-gold howler monkeys (*A. caraya*), black-tufted marmosets (*Callithrix penicillata*) and other vertebrates including pale-throated three-toed sloths (*Bradypus tridactylus*), rodents (*Proechimys* spp.), and birds (Fringillidae, Thaurapidae, Columbidae) [100].

### O’nyong’nyong virus

O’nyong’nyong virus (*Alphavirus*), the agent of o’nyong’nyong fever, is endemic in sub-Saharan Africa and was first isolated during an epidemic in Uganda in 1959 [102]. It is unique among mosquito borne alphaviruses as it is transmitted by night-feeding African anopheline mosquitoes (*Anopheles funestus* and *Anopheles gambiae*). The vertebrate host in the enzootic cycle is unknown; however, mandrills in Gabon (4/25; 16%) have been found seropositive to ONNV by PRNT [102].

### Spondweni virus

Spondweni virus (SPONV) (*Flavivirus*) is closely related to ZIKV and is the agent of Spondweni fever in people in Africa, which is readily confused with Zika [103]. It has been isolated from several species of mosquito, most significantly from the African forest-dwelling *Aedes circumluteolus* [94], but the vertebrate host is unknown. Although previously not thought to exist outside of Africa, SPONV was recently isolated from *Culex* spp. mosquitoes collected in Haiti in 2016 [103].

### Lumbo virus

Lumbo virus (LUMV) (bunyavirus) [47] was isolated from *Aedes pembaensis* in Mozambique 1959 and 1960 during investigations into arboviruses in people. Although people have been found to have neutralizing antibodies against the virus, it is not known to cause clinical disease. Experimental infections of three individual NHPs, a vervet monkey (*Cercopithecus aethiops pygerythrus*), yellow baboon (*Papio cynocephalus*) and bush baby (*Galago crassicaudatus*), resulted in seroconversion and significantly viraemia in the vervet monkey, but no clinical illness [104]. Oddly, outside of Africa, 14/115 (12%) toque macaques in Thailand had LUMV neutralising antibodies [5].

## Conclusions

The literature reviewed in this paper shows that: (i) there are many methods that can be used to identify NHPs and mosquitoes that are important in sylvatic cycles; (ii) there is clear evidence that sylvatic cycles occur in Africa and Asia; mostly these involve NHPs but the role of other forest-dwelling animals must not be overlooked; (iii) there are other arboviruses which might pose future threats to global public health and which might occur in sylvatic cycles with NHPs.

## Data Availability

Not applicable.

## Abbreviations

CHIKV: chikungunya virus
CFT: complement fixation test
DENV: dengue virus
ELISA: enzyme-linked immunosorbent assay
HAI: haemagglutination inhibition
IgG: immunoglobulin G
IgM: immunoglobulin M
IFA: immunofluorescence antibody test
JEV: Japanese encephalitis virus
LUMV: Lumbo virus
MAYV: Mayaro virus
NHP: non-human primate
ONNV: o’nyong’nyong virus
OROV: Oropouche virus
PRNT: plaque reduction neutralisation testing
PCR: polymerase chain reaction
SN: serum neutralization
SPOV: Spondweni virus
VI: viral isolation
ZIKV: Zika virus
